# PDA: an automatic and comprehensive analysis program for protein-DNA complex structures

**DOI:** 10.1186/1471-2164-10-S1-S13

**Published:** 2009-07-07

**Authors:** RyangGuk Kim, Jun-tao Guo

**Affiliations:** 1 Department of Bioinformatics and Genomics, College of Computing and Informatics, University of North Carolina at Charlotte, 9201 University City Blvd, Charlotte, NC 28223 USA

## Abstract

**Background:**

Knowledge of protein-DNA interactions at the structural-level can provide insights into the mechanisms of protein-DNA recognition and gene regulation. Although over 1400 protein-DNA complex structures have been deposited into Protein Data Bank (PDB), the structural details of protein-DNA interactions are generally not available. In addition, current approaches to comparison of protein-DNA complexes are mainly based on protein sequence similarity while the DNA sequences are not taken into account. With the number of experimentally-determined protein-DNA complex structures increasing, there is a need for an automatic program to analyze the protein-DNA complex structures and to provide comprehensive structural information for the benefit of the whole research community.

**Results:**

We developed an automatic and comprehensive protein-DNA complex structure analysis program, PDA (for *p*rotein-*D*NA complex structure *a*nalyzer). PDA takes PDB files as inputs and performs structural analysis that includes 1) whole protein-DNA complex structure restoration, especially the reconstruction of double-stranded DNA structures; 2) an efficient new approach for DNA base-pair detection; 3) systematic annotation of protein-DNA interactions; and 4) extraction of DNA subsequences involved in protein-DNA interactions and identification of protein-DNA binding units. Protein-DNA complex structures in current PDB were processed and analyzed with our PDA program and the analysis results were stored in a database. A dataset useful for studying protein-DNA interactions involved in gene regulation was generated using both protein and DNA sequences as well as the contact information of the complexes. WebPDA was developed to provide a web interface for using PDA and for data retrieval.

**Conclusion:**

PDA is a computational tool for structural annotations of protein-DNA complexes. It provides a useful resource for investigating protein-DNA interactions. Data from the PDA analysis can also facilitate the classification of protein-DNA complexes and provide insights into rational design of benchmarks. The PDA program is freely available at .

## Background

**Figure 1:**
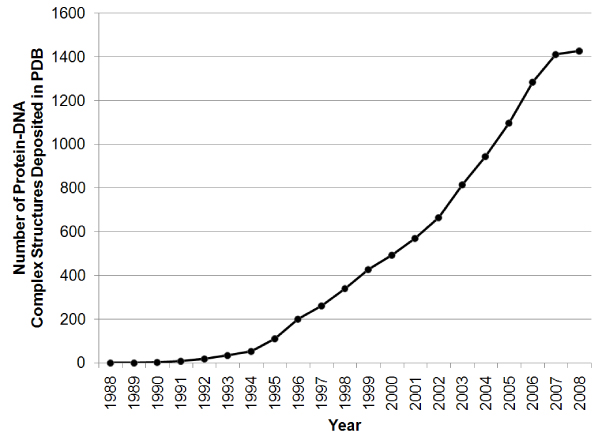
**Trend of the number of pr otein-DNA complexes in Protein Data Bank (PDB)**.

Protein-DNA interactions play crucial roles in many biological processes, including regulation of gene expression, DNA modification, and DNA duplication [1]. Knowledge of the 3-dimensional (3D) structures of protein-DNA complexes can help us better understand the mechanism of protein-DNA recognition, shed light on the evolution of gene regulatory networks, and guide the rational design of therapeutic drugs. With the advancement of structure determination techniques and molecular expression systems, the number of protein-DNA complex structures deposited in Protein Data Bank (PDB) [2] is increasing at a higher rate. As of August, 2008, there are over 1400 solved protein-DNA complex structures in PDB (Figure 1). The current state of research in protein-DNA interactions with the number of known protein-DNA complex structures is reminiscent of the situation of protein structure modeling in the early 1990s which started a new wave of development of protein structure prediction methods. The number of available high-resolution structures of protein-DNA complexes makes it possible to develop more accurate knowledge-based potentials and protein-DNA docking methods [3-5].

**Figure 2:**
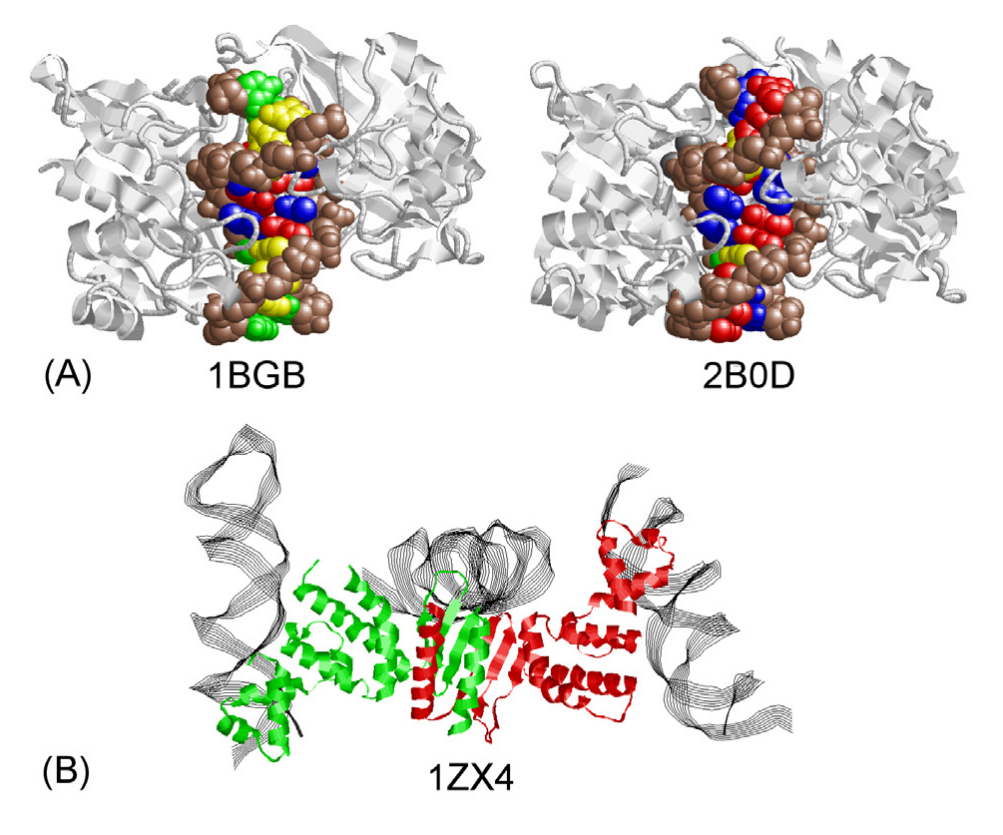
**Examples of different double-stranded DNAs bound to the same protein**. (A) Protein-DNA complexes that have similar proteins but different DNA molecules (PDB IDs 1BGB and 2B0D). Adenines, thymines, guanines, cytosines, and backbone atoms are colored in red, blue, yellow, green, and brown, respectively. (B) Sequence alignment between the DNA sequences of 1BGB and 2B0D. (C) The dimer of P1 ParB fragments of plasmid partition par B protein (1ZX4) has two different DNA binding domains.

One of the crucial steps in investigating the mechanism of protein-DNA interactions is the construction of datasets as well as classification of protein-DNA complex structures. Previously, non-redundant datasets have been generated through comparison of protein sequences [6-11]. This "protein-centric" view has also been the traditional method in classification of protein-DNA complexes [12]. However, the same protein may interact with two very different DNA molecules. For example, 1BGB and 2B0D contain the same protein but the corresponding DNA sequences (GGGATATCCCG and AAAGAATTCTTT) are very different (Figure 2A and 2B). In addition, one protein may interact with two DNA sequences through different binding sites. The protein-DNA complex structure 1ZX4 is such an example, in which the protein has two different DNA binding domains (Figure 2C). One of the reasons that the DNA molecules were not taken into account in dataset construction is probably the lack of double-stranded DNA sequence information and complete protein-DNA binding models in PDB files. For example, some double-stranded DNA molecules are fragmented into several shorter DNA chains for biological or non-biological reasons, and there is no annotation about whether a DNA chain is a single stranded DNA, one full-length chain of a double-stranded DNA, or only a fragment of one strand of a double helix. Moreover, DNA bases that interact with protein residues are usually more conserved than other bases. Therefore, how the DNA sequences in the complexes are used for comparison is not a trivial issue. The lack of such detailed information has compromised the classification of protein-DNA complexes and dataset construction.

Due to the unique structural features of DNA, protein residues may interact with DNA bases in major or minor grooves, and the protein-DNA interactions can be specific or non-specific. Although these features are important in characterizing the nature of the interactions in a protein-DNA complex, they are not available in PDB files. Currently, there are several programs and databases, such as 3DNA [13,14], Nucleic Acid Database (NDB) [15], Amino Acid-Nucleotide Interaction Database (AANT) [16], and Protein-Nucleic Acid Complex Database (ProNuC) [17], which represent previous efforts in providing some structural details of DNA or protein-DNA complex structures. However, these programs/databases only provide information on some aspects of the protein-DNA complex structures. For example, NDB and 3DNA are nucleic acid specific. AANT only has statistical information on amino acid-nucleotide interaction. While ProNuC provides a list of atom-atom contact pairs between protein and DNA, it lacks other information such as the nature of protein-DNA interactions.

Sarai and colleagues recently developed a new scheme for classification of protein-DNA complexes using a "DNA-centric" approach [12,18]. The new viewpoint highlights the need for a comprehensive annotation of the solved protein-DNA complex structures and an automatic program for generating such information. Here we present the development of such a program, PDA (for *p*rotein-*D*NA complex structure *a*nalyzer), which can help us better understand the mechanism of protein-DNA interactions and should be useful in statistical potential development, protein-DNA docking, and structure-based regulatory network studies. In addition, the protein-DNA complex structures can be classified from a more holistic view by combining the "protein-centric" and "DNA-centric" approaches.

## Methods

**Figure 3:**
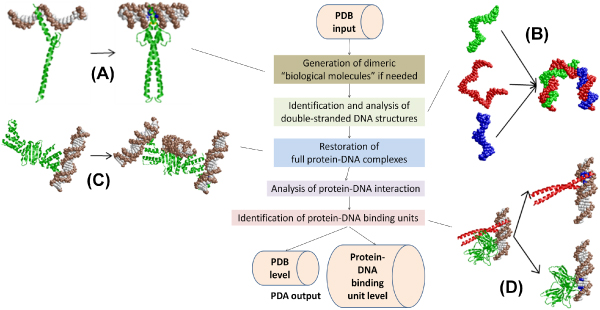
**Flowchart of PDA analysis**. (A) Restoration of "biological molecules" (1AN2); (B) Reconstruction of double-stranded DNAs (2IIE); (C) Reconstruction of missing double-stranded DNAs (1ZX4); and (D) Identification of protein-DNA binding units (1A02).

PDA is implemented using Python [19], a platform-independent programming language. The flowchart of PDA is shown in Figure 3, which includes four major steps: 1) restoration of full protein-DNA complex structures (Figure 3A and 3C); 2) DNA structure analysis including identification of base pairs and double-stranded DNA (Figure 3B); 3) analysis of protein-DNA interactions; 4) identification of protein-DNA binding units (Figure 3D). Below we provide detailed description for each step.

### Restoration of full protein-DNA complex structures

Some PDB entries provide only partial coordinates of the whole protein-DNA complex structures. We observed that there were two kinds of incompleteness of the protein-DNA complex structures in PDB files. The first is that parts of the complex structures, such as one chain of a double-stranded DNA or one chain of a protein dimer is missing in the original PDB file (Figure 3A). PDB files with this type of incomplete structure usually have codes, e.g. "biological molecules", embedded in the structure file, which PDA uses for generating the full structure model if missing component(s) is identified. The second type of incomplete complex structures is that the coordinates of one or more full double-stranded DNAs are missing (Figure 3C). PDA searches for such missing double-stranded DNAs by first reconstructing 3 × 3 crystal cells with the crystal symmetry information of the structure in PDB and examining if there is any double-stranded DNA in the crystal whose bases are in contact with the protein(s).

### DNA structure analysis

#### Base-pair and double-stranded DNA detection

**Figure 4:**
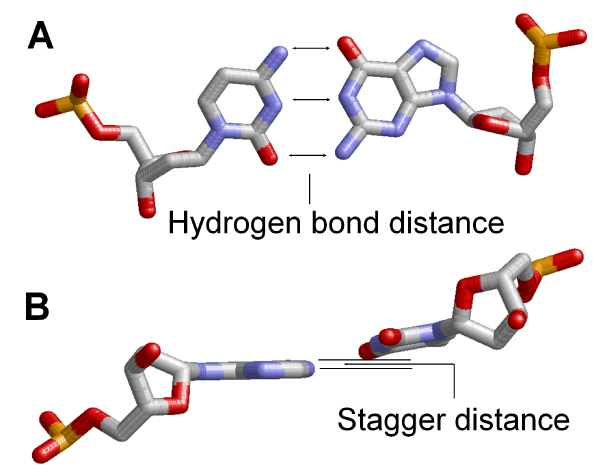
**Schematic representation of DNA base-pair detection by PDA**.

Many algorithms have been developed for defining base-pairs in a DNA structure [13,20-25]. However these programs employ complicated procedures for base-pair recognition. Since the goal of this step is to identify double-stranded DNAs for DNA sequence comparison, we developed a simple approach for detecting base pairs by using two distance measures: "H-distance" and "stagger distance". H-distance is the distance between a hydrogen bond donor of one base and its hydrogen bond acceptor of the other base (Figure 4A) while stagger distance represents the distance between the plane of one base and the tip heavy atom of the other base (Figure 4B). Therefore, one base-pair has three (for C-G) or two (for A-T) H-distances and two stagger distances. A base-pair is defined between two bases when both the maximum H-distance and the maximum stagger distance are less than their respective cutoff values. If a base has more than one potential pairing partner, the one with the smallest stagger distance will be chosen. Using a heuristic approach, we found that a combination of an H-distance cutoff of 4.5 Å and a stagger distance cutoff of 1.5 Å can accurately identify structural base pairs. A double-stranded DNA is then defined as a group of polynucleotide chains that are canonically joined by base-pairing (for example, a holiday junction is not considered as a double-stranded DNA). DNA chains without any base pairing partners are classified as single-stranded DNAs. As an optional feature of PDA, the users can also use 3DNA instead of the default functional module in PDA for base pair detection.

#### Recognition of DNA bending with a backbone break

**Figure 5:**
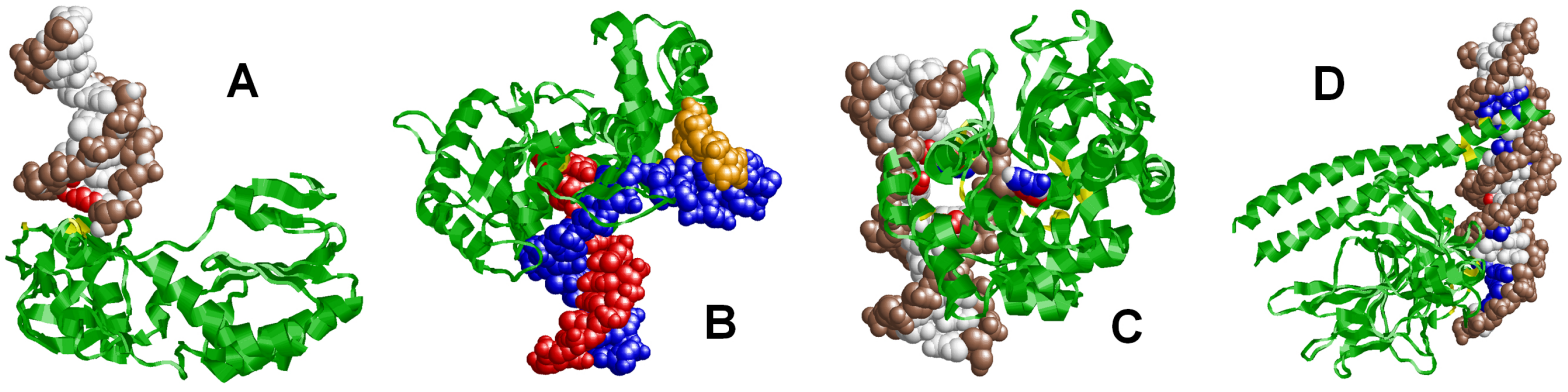
**Protein-DNA complexes with special features**. Protein-DNA complex structures with (A) running-into-protein DNA, moloney murine leukemia virus reverse transcriptase-DNA complex (1D1U); (B) a crack in a double-stranded DNA, human DNA polymerase beta-DNA complex (1BPZ), DNA chains were colored to make the crack in the double helix more visible; (C) a flipped-out base, 8-oxoguanine DNA glycosylase-DNA complex (2NOB), and (D) multiple protein binding sites; NFAT and Jun and Fos bound to DNA (1A02). Default color scheme for displaying protein-DNA complex structures are: Blue (DNA major-groove atoms in contact with protein), Red (DNA minor-groove atoms in contact with protein), Brown (DNA backbone atoms), White (non-protein-contacting DNA bases), Yellow (protein residues in contact with DNA), and Green (non-DNA-contacting protein residues).

Binding to proteins can cause DNA deformation and introduce bends in DNA molecules. Severe deformation with broken DNA backbones has been observed in protein-DNA complex structures that are not related to gene regulation (Figure 5B). We developed an empirical method to detect such a "crack" in a DNA backbone: when one or more sequential nucleotides are missing in one strand of a double-stranded DNA, the distance between the C_1_' atoms of the nucleotides flanking the break or missing region is calculated. If the distance is more than (*d *× 6 + 3) Å, where *d *is the sequence distance between the flanking nucleotides, a crack is recorded. Most of the cracks detected by this criterion were related to DNA modification and replication (data not shown).

### Protein-DNA interaction analysis

A DNA base is considered to be in contact with a protein if the distance between any heavy atom of the base and any heavy atom of the protein is less than a cutoff value (the default cutoff is 4.8 Å in PDA). If the contact involves a base in the major/minor groove, it is annotated as a major/minor groove contact. When the distances between both the major and minor groove atoms of a base and a protein atom are within the cutoff value, the type of the base-protein contact is determined by comparing the contact distances and the angle formed by the "major groove atom"-"protein atom"-"minor groove atom". If the angle is less than 40 degrees, the contact with the longer distance is considered to be shielded by the shorter contact and is thus discarded. In case that the angle is more than 40 degrees, both the major and minor groove atoms of the base are considered to be in contact with the protein atom, which was usually observed in terminal bases and the bases that do not have base-pairing partners (for example, the DNA glycosylase-DNA complex shown in Figure 5C). We use several measures to describe the nature of protein-DNA interactions: 1) major (minor) groove contact number refers to the number of major (minor) groove DNA bases that are in contact with protein; 2) major groove contact ratio is calculated as the ratio between the number of major groove contacts and the sum of major and minor groove contacts; 3) base (backbone) contact number refers to the number of nucleotides whose base (backbone) is in contact with protein; 4) base contact ratio is calculated as the ratio between the number of base contacts and the sum of base and backbone contacts. Additional aspects of protein-DNA interaction that are analyzed by PDA include "running-into-protein" DNA (when the axis of a double-stranded DNA is blocked by a protein) (Figure 5A) and "flipped base" (if a base in a double-stranded DNA does not have a base-pairing partner and is in contact with protein) (Figure 5C).

### Protein-DNA binding unit

**Figure 6:**
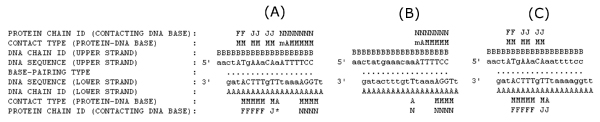
**A sample PDAgram for **1A02. (A) Two protein complexes, NFAT, and Fos-Jun, bind to different sites on the double-stranded DNA of protein-DNA complex structure 1A02. PDA identifies and separates the protein-DNA binding units as in (B) the NFAT-DNA complex and (C) the Fos-Jun-DNA complex. Protein chain N, F, and J are for NFAT, Fos, and Jun, respectively. A dot (.) represents a canonical base pair. An uppercase-case letter in a DNA sequence represents the base is in contact with the protein while a lower-case letter is not. 'M', 'm', or 'A' above or below a DNA chain indicates that the interaction between the protein and the base is in the major, minor, or both major and minor grooves. '*' above or below a DNA base means more than one protein chain is in contact with the DNA base.

A PDB entry can have more than one protein-DNA binding sites; for example, 1A02 has two distinct protein-DNA binding entities on one double-stranded DNA (NFAT-DNA and FOS-JUN-DNA) (Figures 3D and 6). Comparison of such complexes can be problematic as these complexes have different protein-DNA binding entities. To resolve such a problem, we use a new term "protein-DNA binding unit" to describe the distinct interaction unit: a double-stranded DNA and a functional protein entity (one protein chain or interacting chains) bound to the DNA. As in the case of 1A02, it has two DNA-binding protein components that do not interact with each other, therefore it is considered to have two protein-DNA binding units (Figure 6).

### Functional classes of protein-DNA complexes

Each protein-DNA complex structure is assigned with one of four functional classes ("gene regulation", "transferase", "hydrolase" and "others") based on the keywords in the PDB file of a protein-DNA complex structure. Entries with "transcription" or "gene regulation" as keywords belong to the gene regulation class. The transferase class contains structures with keywords "transferase" or "polymerase" while the hydrolase class consists of PDB entries with annotated function of "hydrolase" or "nuclease". The protein-DNA complex structures that cannot be assigned with any of these three classes are grouped into the "others" category. In case of conflicts, the function of the complex structure is further examined by manual inspection. For example, a few PDB entries have keywords for both the transferase class and the gene regulation class. All of them were classified as transferase after manual inspection.

### Sequence comparison of protein-DNA complex structures

Sequence comparison is a convenient way for determining the similarity of two macromolecules such as two protein or two DNA sequences. It would be useful if such sequence comparison could also be done for protein-DNA complexes. Previous studies only compare protein sequences for dataset construction. Since a protein-DNA complex can have multiple protein chains as well as multiple double-stranded DNAs, we take an approach of all-against-all comparison (protein vs. protein and DNA vs. DNA) of two complexes and report the lower and upper bounds of the sequence identities for protein and DNA separately. While the sequences of the entire protein chains are used for protein comparison, the DNA sequences used for comparison are not straightforward. Some protein complexes have long DNA sequences but only a small portion of the sequences are involved in protein-DNA interaction. On the other hand, in some protein-DNA complexes, a large percentage of DNA participates in the binding and interaction with proteins even though the DNA sequences are short. To address this issue, we first extract the DNA subsequences that interact with proteins since in general the DNA binding motifs are better conserved while the flanking sequences showed less conservation. The protein-binding DNA fragment is defined as the longest DNA subsequence bounded by two bases that are in contact with the protein plus one flanking base on each side (5' and 3'). Within the subsequence, at most three consecutive bases are allowed to be not in contact with the protein. If there is no base-protein contact in a double-stranded DNA, the double-stranded DNA is excluded from sequence comparison. Likewise, protein chains that are not in contact with any bases of DNAs are also excluded from sequence comparison. ALIGN [26,27] is used for protein sequence comparison, with gap opening and extension penalty of -12 and -2, respectively. As for the DNA sequence comparison, we used an in-house program to perform gapless alignments since the binding motifs are generally short. Sequence identity is defined as the number of identical residues or bases in the alignment divided by the length of the shorter sequence.

## Results and discussion

### Performance of base-pairing detection by PDA

To test the efficiency of PDA that uses only two distances (H-distance and stagger distance) for base-pair detection, we compared the performance of PDA with 3DNA [13], a program widely-used for DNA structure analysis, on a dataset of 1077 protein-DNA complex structures that are solved by X-ray crystallography with high resolution (less than 3.5 Å) and have at least one base-pair determined by 3DNA. Two base-pairing matrices were generated for each DNA by PDA and 3DNA respectively. Each cell has a value of 1 if two bases form a pair and 0 otherwise. The correlation of base-pair assignments between PDA and 3DNA was calculated using Matthews Correlation Coefficient (MCC) [28]. The histogram of the MCC for the 1077 protein-DNA complex structures is shown in Figure 5. The MCC of more than 99% of the complexes is more than 0.90 and 73% of the complexes show a perfect correlation between 3DNA and PDA. Compared with the 3DNA assignment, most of the missed base-pairs by PDA were located at the termini of DNA or in the middle of very long and wound DNA. There are some base-pairs detected only by PDA but not 3DNA. Through manual inspection, we found that many of these "false positive" base-pairs are possibly true base-pairs. Based on above analysis, the performance of PDA in base-pair detection is comparable to that of 3DNA. Our simple but effective approach uses less than five distance calculations per base-pair while 3DNA employs a least square fitting procedure to obtain a reference frame for each base followed by comparing six geometrical parameters from two reference frames for a pair of bases.

### PDA analysis of protein-DNA complex structures

PDA takes a PDB file as input and outputs the detailed analysis result to the standard output as well as files for protein-DNA binding units. Most of the PDA output is self-explanatory. Several notable features of PDA are as follows. One is the PDAgram, a text-based diagram from PDA analysis showing the organization and structure of double-stranded DNAs and the interaction patterns between protein and double-stranded DNA (Figure 6). The advantage of PDAgram over 3D visualization of protein-DNA complexes is that it provides an easy way to display the interaction pattern of a protein-DNA complex. For 3D visualization of PDA analysis data, a RasMol/Jmol [29,30] visualization script is automatically created for each PDA analysis report, in which the protein and DNA are rendered in cartoon and space-fill formats, respectively, with a default color scheme as shown in Figure 5D.

### Structural features of protein-DNA complex structures by PDA analysis

**Figure 7:**
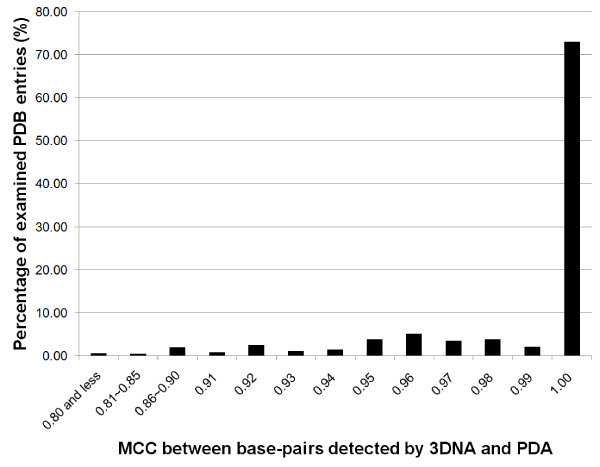
**Histogram of the MCC correlations between base-pair detection by 3DNA and PDA**.

PDA has the capability to restore full protein-DNA complex structures (Figure 3A and 3C), recognize double-stranded DNA structure, reconstruct the full-length double-stranded DNA (Figure 3B), and identify protein-DNA binding units (Figure 3D and 7). Figure 3C shows the utility of PDA's complex structure restoration capability. The PDB file of 1ZX4 has the coordinates for one protein dimer and one double-stranded DNA. However, in the original literature for 1ZX4[31], three copies of the double-stranded DNA were shown to bind to the same protein dimer (on two different DNA binding domains). Using the crystal symmetry information, PDA reconstructed the coordinates of the whole protein-DNA complex structure of 1ZX4 as reported [31]. The PDA program is also capable of detecting protein-DNA complexes that have running-into-protein DNA (Figure 5A), cracks in a double-stranded DNA (Figure 5B), and flipped-out bases as described in Methods (Figure 5C). Running-into-protein DNAs and flipped-out bases are often observed in reverse transcriptase-DNA complexes and DNA modifying enzyme-DNA complexes, respectively.

**Figure 8:**
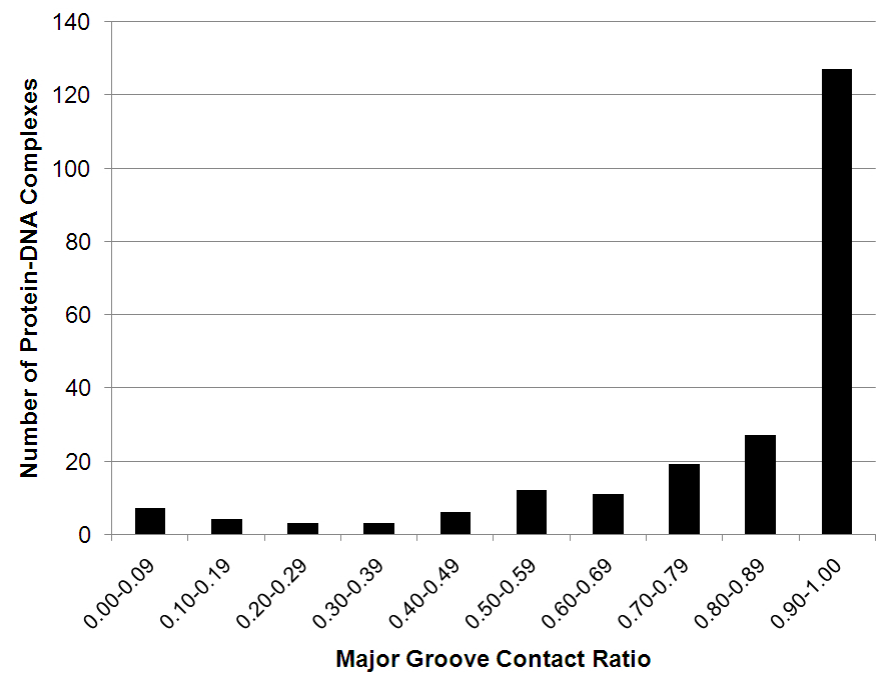
**Major groove contact ratio in a non-redundant dataset of protein-DNA complex structures**.

Major/minor groove-protein contacts are important in studying protein-DNA recognitions. Figure 8 shows the distribution of major groove contact ratios by PDA analysis of 219 non-redundant protein-DNA complex structures chosen from PDB with the following criteria: 1) solved by X-ray crystallography with a resolution of at least 3.5 Å; 2) at least one base-protein contact; and 3) each pair in the dataset has less than 30% protein sequence identity. We found that about 10.5 percent (23 out of 219) of the complexes have more minor groove-protein contacts than major groove-protein contacts suggesting that minor groove-protein contacts may play important roles in protein-DNA binding specificity in a number of protein-DNA complexes [32,33].

### Clustering of protein-DNA complex structures involved in gene regulation

**Figure 9:**
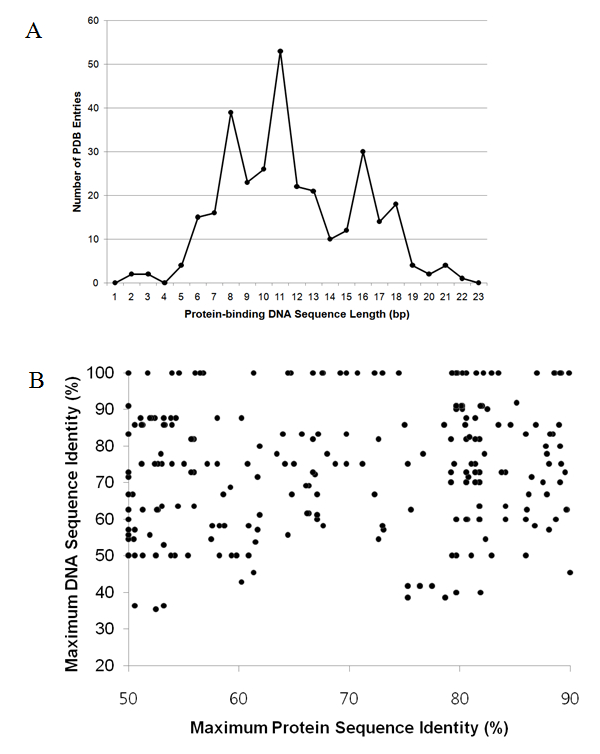
**DNA sequence length and similarity analysis in Set263**. (A) Length distribution of protein-interacting DNA sequences; (B) DNA sequence similarities in protein-DNA complexes with more than 50% protein sequence identity.

To further demonstrate PDA's utility in studying systems biology and structure-based transcription factor binding site prediction, we generated a non-redundant dataset with protein-DNA complex structures that are involved in gene regulation. First, a total of 1307 protein-DNA complex structures that were solved using X-ray crystallography with resolutions less than 3.5 Å were selected. We then selected the protein-complex structures that are in the "gene regulation" category and applied the following criteria: 1) the number of protein-DNA binding units ≥ 1; 2) base contact ratio ≥ 0.3 to ensure specific interactions; 3) base contact number ≥ 2; 4) no running-into-protein double-stranded DNA; 5) no "crack" in double-stranded DNA structures. Application of the above procedure resulted in 266 complex structures. The length distribution of the protein-contacting DNA sequences as described in Methods and Implementation is shown in Figure 9A. The length of most of the DNA sequences ranges from 6 to 18 base pairs. The complex structures with DNA shorter than 6 bps and longer than 18 bps were removed and a set of 263 (Set263) complex structures were generated for further studies.

Since the DNA sequences involved in protein-DNA interaction are generally short (Figure 9A), two unrelated DNA sequences may have high sequence similarity. In Set263, there are 49874 complex pairs in which the proteins have less than 30% sequence identity. When the corresponding DNA sequences were compared, we found that about 93% of the DNA sequences have up to 65% sequence identity even though the protein sequences are not similar (data not shown). It is not surprising that all the DNA sequence pairs showed at least 25% sequence identity using gapless sequence alignment approach as the DNA sequences are short. On the other hand, there are 252 pairs of protein-DNA complexes that have less than 65% DNA sequence identity while the proteins have more than 50% sequence identity (Figure 9B).

In general there is a trade-off between "redundancy" and "dataset size" for statistical analysis when constructing a dataset especially if the data available is not large enough as in the case of protein-DNA complex structures. For example, when only protein sequences are used for protein-DNA complex comparison, a low sequence identity cutoff (e.g. 25%) will generate a relatively small dataset. This dataset offers low-redundancy but lacks power in statistical analysis [5]. While a higher protein sequence identity cutoff increases the dataset size, the "non-redundancy" is compromised. Note that protein-DNA complexes may have dissimilar DNA sequences and interaction patterns even though the protein sequence identity is over 50% (Figure 3 and Figure 9B) [4]. Therefore, it is possible to produce a dataset that is bigger while keeping a low data redundancy in terms of the nature of protein-DNA interactions by increasing the cutoff of protein sequence similarity and applying DNA sequence similarity at the same time. As an application example, we clustered complex structures in Set263 into 104 groups using a sequence identity cutoff of 50% for both the protein and double-stranded DNA. Non-redundant datasets can be selected from the 104 distinct clusters and used for studying transcription factor-DNA interactions. To our knowledge, this is the first attempt that not only considers the number of base-protein contacts, ratio of specific contacts between protein and DNA but also take the double-stranded DNA sequence identity into account. These datasets are available at .

## Conclusion

We developed an automatic and comprehensive analyzer for protein-DNA complex structures and implemented it as a computer program PDA. PDA can restore the full atomic coordinates of protein-DNA complex structures from partial coordinates, accurately detect DNA base-pairs with a new and simple algorithm, recognize double-stranded DNA structures, analyze protein-DNA contacts and define protein-DNA binding sites. These restorations and annotations are necessary for constructing datasets that takes the DNA into consideration, making them real non-redundant "complex structures", not just non-redundant in terms of proteins. PDA's analysis on protein-DNA binding modes, including major/minor groove interactions and base/backbone-protein contacts, will also help classification of protein-DNA complex structures and construction of contact specific datasets for protein-DNA interaction studies.

## Availability and requirements

PDA and pre-compiled PDA analysis results for protein-DNA complex structures in PDB are freely available for non-commercial use at . The only requirement for running PDA is a Python interpreter (tested on Python v2.4.2). Java virtual machine, which is available free at , is required for using the precompiled analysis data at . The webserver was successfully tested with FireFox 2, Safari 3 and Internet Explorer 6. The PDA program and web server will be updated regularly.

## Competing interests

The authors declare that they have no competing interests.

## Authors' contributions

JG designed and supervised the study and revised the manuscript. RGK participated in the design of the study, implemented the computer program and the web server, and prepared the manuscript. All authors read and approved the final manuscript.
